# Supplementary material for: The Effect of Mono- and Di-Saccharides on the Microbiome of Dairy Cow Manure and Its Odor
Source: Microorganisms. 2024 Dec 31;13(1):52. doi: 10.3390/microorganisms13010052 (PMC11767979; doi:10.3390/microorganisms13010052)
Supplement: Supplementary file 1 [file microorganisms-13-00052-s001.zip › Supplementary Figure Alpha Diversity Bar Plots.pdf]

Supplementary Figures

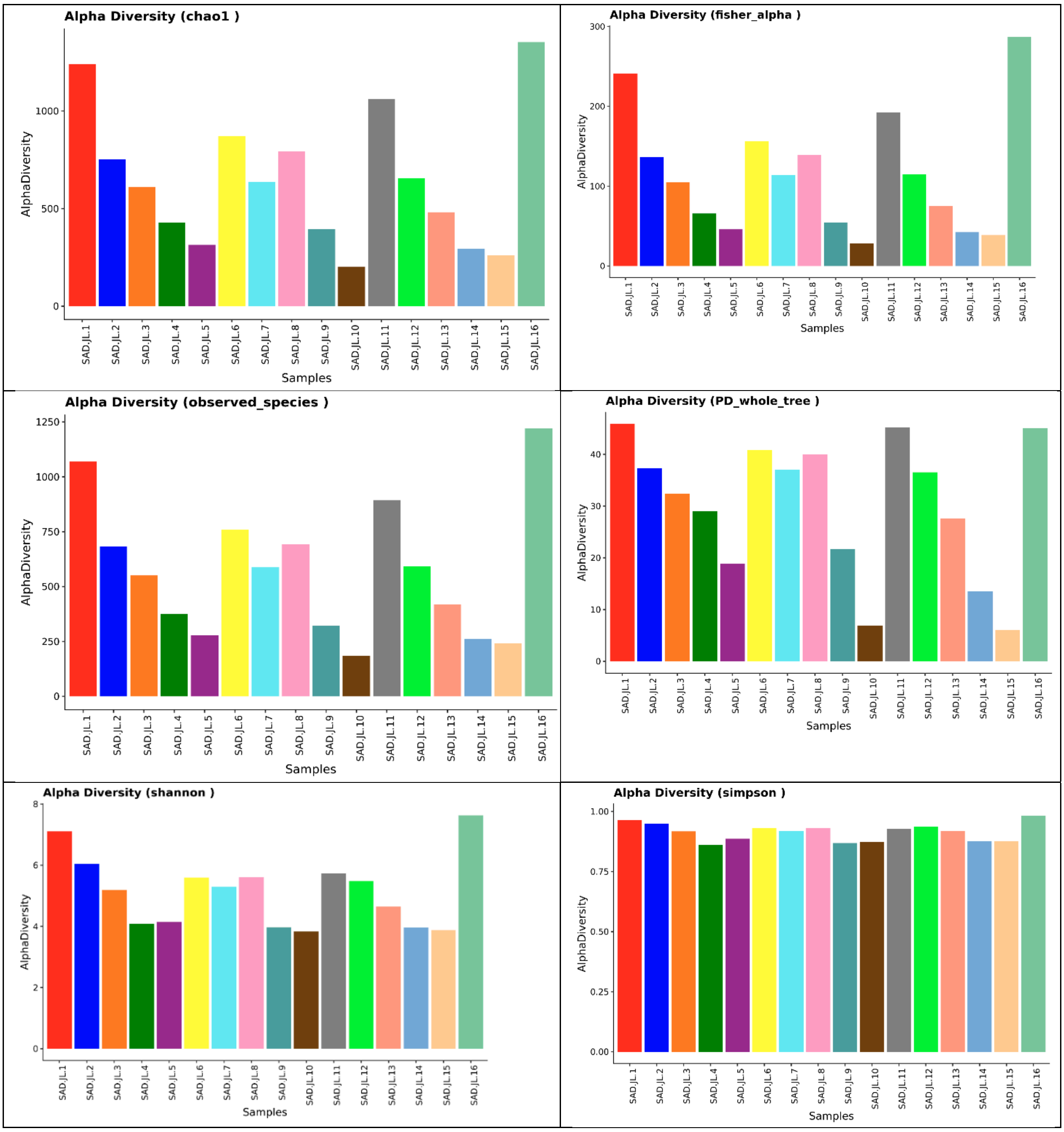

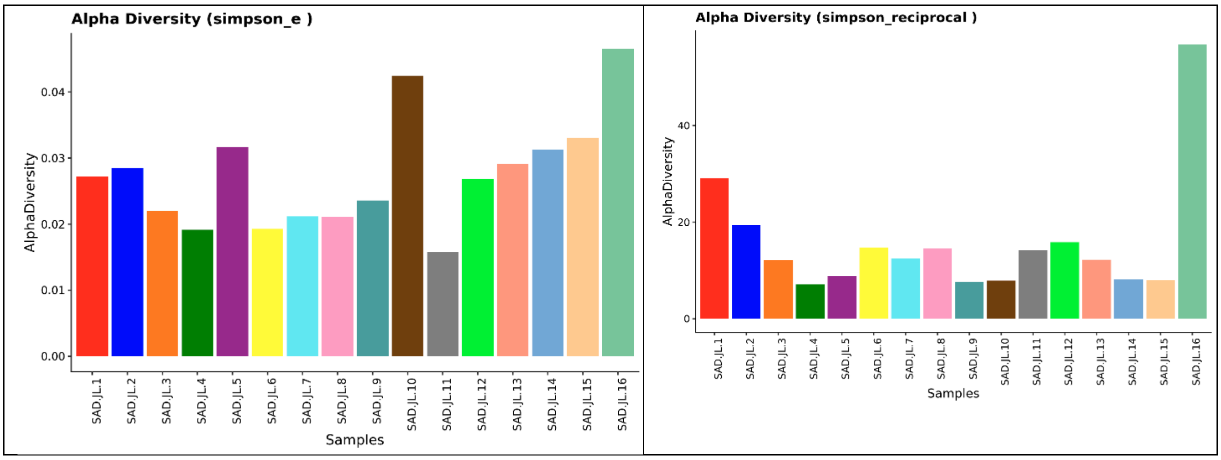

Supplementary Figure S1: The alpha diversity measures at sample level.

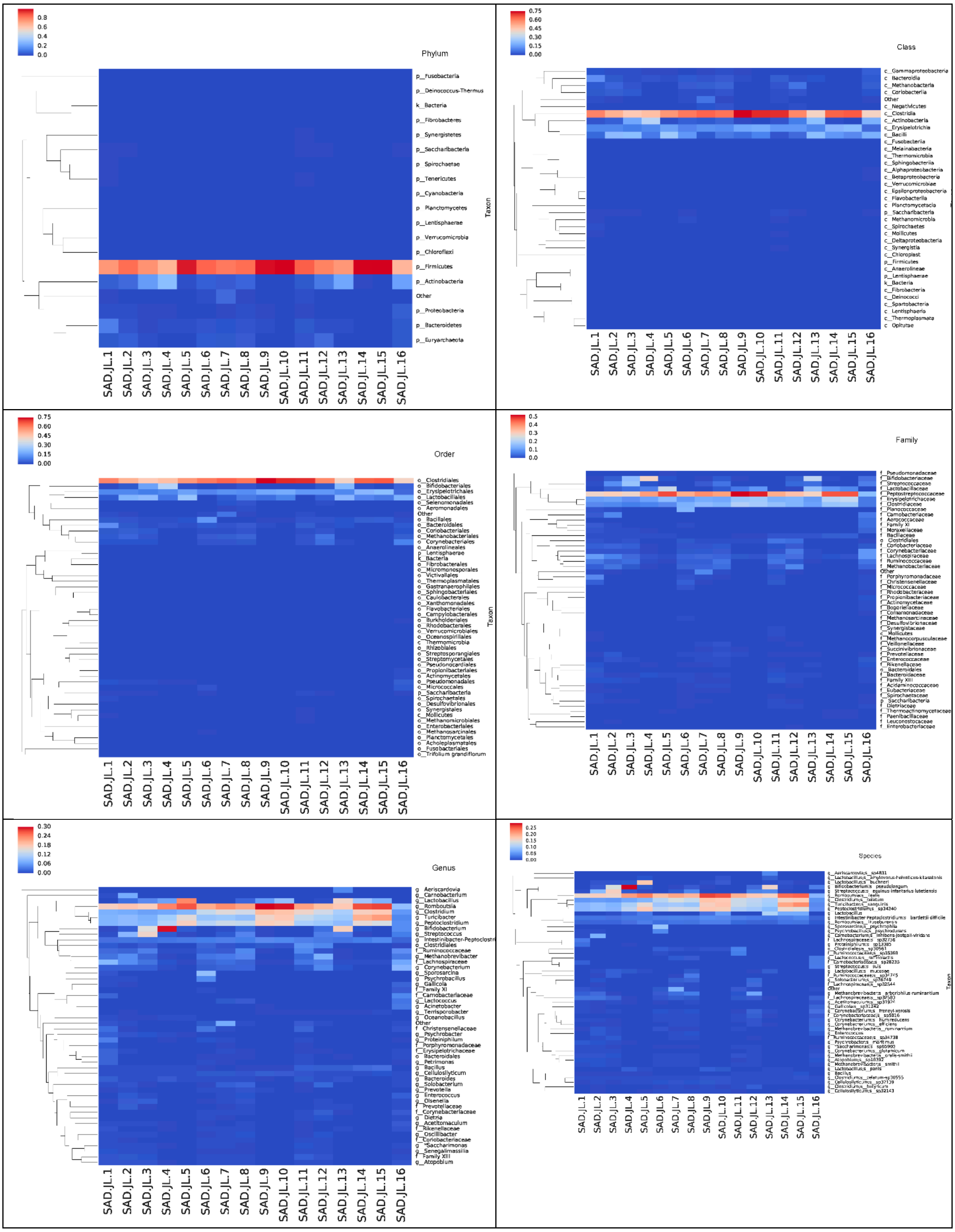

Supplementary Figure S2: Taxonomy abundance without sample clustering. Hierarchical clustering based on Bray-Curtis dissimilarity index was performed on the taxa so that taxa

with similar distributions are grouped together. The heatmap for microbial composition of the samples at the species level shows the top fifty most abundant species identified.
