# Supplementary figures and images for: The Effect of Mono- and Di-Saccharides on the Microbiome of Dairy Cow Manure and Its Odor
Source: Microorganisms. 2024 Dec 31;13(1):52. doi: 10.3390/microorganisms13010052 (PMC11767979; doi:10.3390/microorganisms13010052)

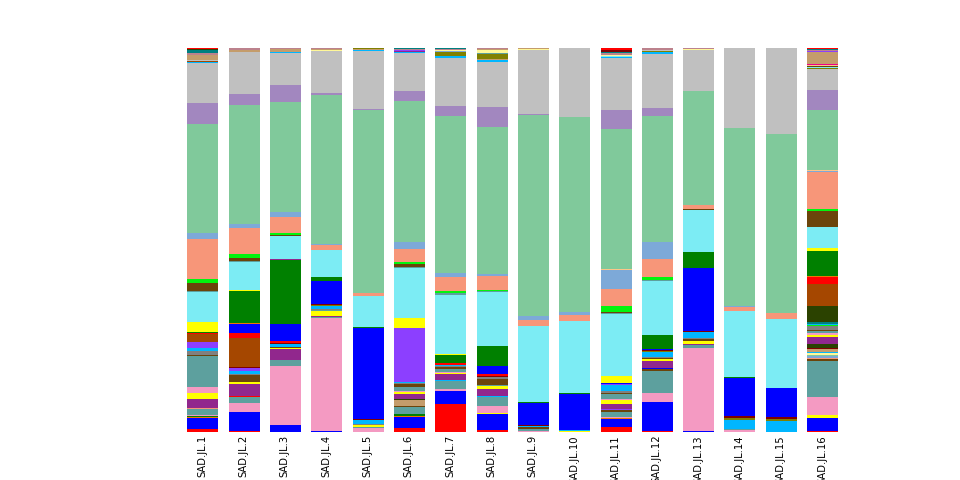

Supplement: Supplementary file 1 [file microorganisms-13-00052-s001.zip › Taxa Summaries_files/266Cn5Q6500HYmxthid1FzKNPAknbz.png]

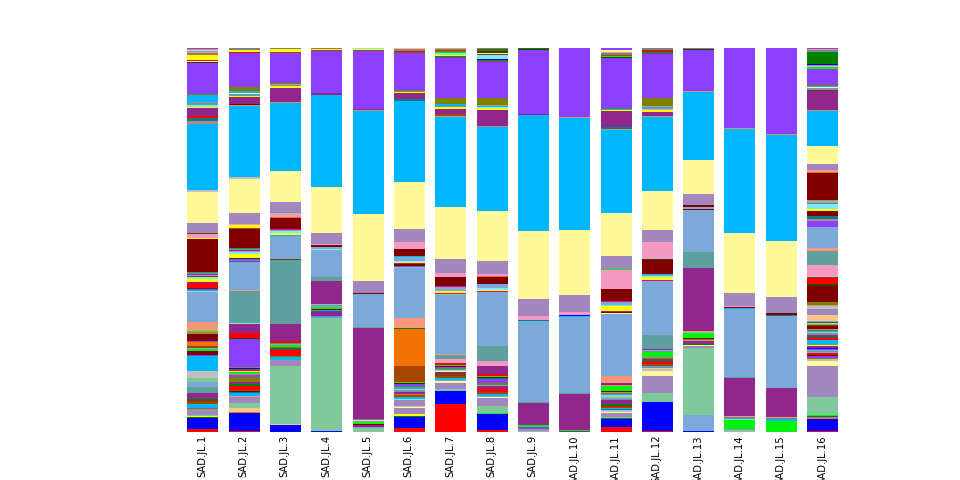

Supplement: Supplementary file 1 [file microorganisms-13-00052-s001.zip › Taxa Summaries_files/8gbeOtBqZWBZ2qSNIhg98SjF1n5aik.png]

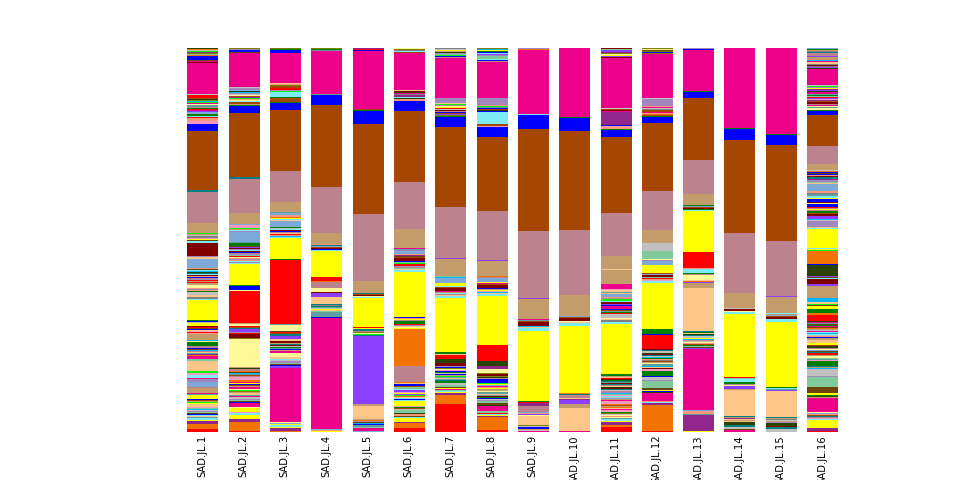

Supplement: Supplementary file 1 [file microorganisms-13-00052-s001.zip › Taxa Summaries_files/AqTpxuxjq2nYaxKEBu8I0ul2obXNF8.png]

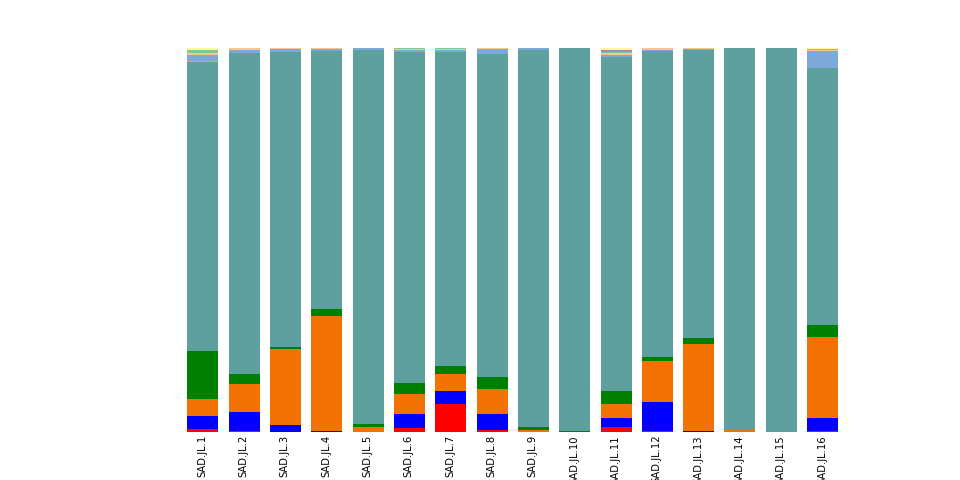

Supplement: Supplementary file 1 [file microorganisms-13-00052-s001.zip › Taxa Summaries_files/H0cKWRldbcm0oE6j80eS2L80Z710dU.png]

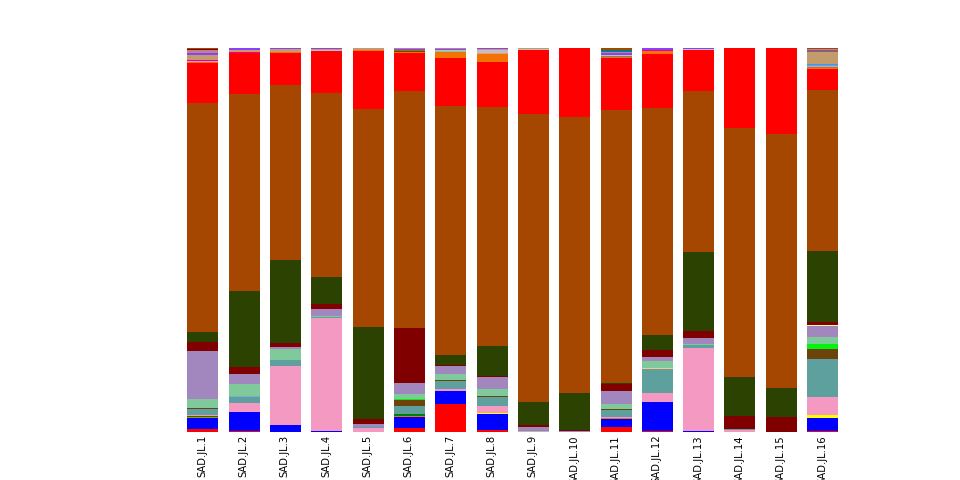

Supplement: Supplementary file 1 [file microorganisms-13-00052-s001.zip › Taxa Summaries_files/Nqrmg2tYouuYOqgzZXhYE05Gz5W00c.png]

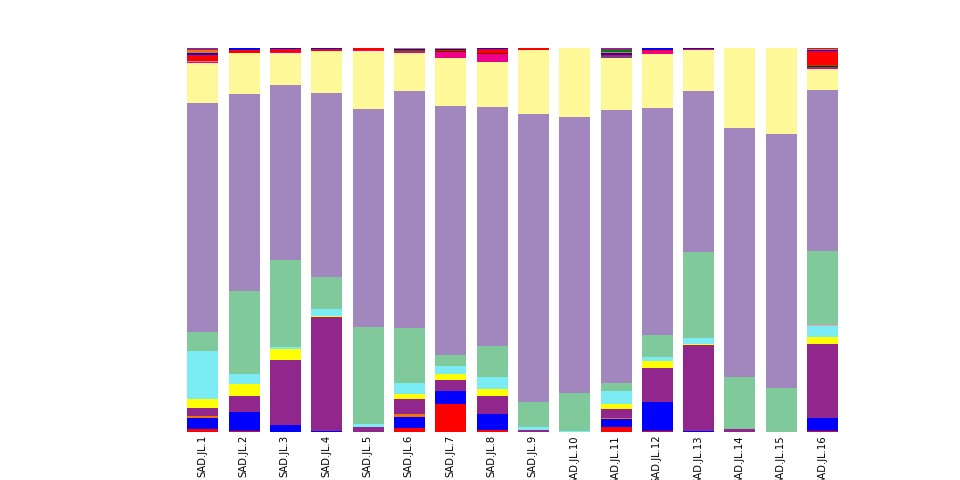

Supplement: Supplementary file 1 [file microorganisms-13-00052-s001.zip › Taxa Summaries_files/oSZeygDSjZhsn1z3yz01dGEb1czFJg.png]
